# Sexual Well-Being in Adult Male Patients with Congenital Adrenal Hyperplasia

**DOI:** 10.1155/2014/469289

**Published:** 2014-02-10

**Authors:** Bogna Dudzińska, Jonas Leubner, Manfred Ventz, Marcus Quinkler

**Affiliations:** Clinical Endocrinology, Charité Campus Mitte, Charité University Medicine Berlin, Charitéplatz 1, 10117 Berlin, Germany

## Abstract

*Introduction.* Men with congenital adrenal hyperplasia (CAH) due to 21-hydroxylase deficiency show impaired fecundity due to testicular adrenal rest tumors and/or suppression of the gonadal axis. Sexual well-being might be an additional factor; however, no data exists. *Patients and Methods.* Prospective longitudinal monocentric study included 20 male CAH patients (14 salt wasting, 6 simple virilizing; age 18–49 yr). Clinical assessment, testicular ultrasound, biochemical and hormonal parameters, three validated self-assessment questionnaires (SF-36, GBB-24, and HADS), and male Brief Sexual Function Inventory (BSFI) were analyzed at baseline and after two years. *Results.* Basal LH and testosterone levels suggested normal testicular function. LH and FSH responses to GnRH were more pronounced in patients with a good therapy control according to androstenedione/testosterone ratio < 0.2. This group had significant higher percentage of patients on dexamethasone medication. GBB-24, HADS, and SF-36 showed impaired *z*-scores and no changes at follow-up. BSFI revealed impairments in dimensions “sexual drive,” “erections,” and “ejaculations,” whereas “problem assessment” and “overall satisfaction” revealed normal *z*-scores. Androstenedione levels correlated (*P* = 0.036) inversely with *z*-scores for “sexual drive” with higher levels associated with impaired “sexual drive.” *Conclusion.* Male CAH patients showed a partly impaired sexual well-being which might be an additional factor for reduced fecundity.

## 1. Introduction 

Fecundity is reduced in male patients with congenital adrenal hyperplasia (CAH) due to 21-hydroxylase deficiency. Recent studies suggested that development of testicular adrenal rest tumors (TARTs), which cause an obstruction of the seminiferous tubules, may play a major role [1, 2]. In addition, suppression of the gonadal axis due to adrenal androgen excess might also cause reduced fertility [2]. Both pathomechanisms are thought to be a consequence of insufficient hormonal control [3].

Besides these somatic causes of impaired fertility in CAH males, there might be aspects of psychosocial adaption and sexual well-being which may be additional factors for impaired fertility. However, up to now there are no studies investigating sexual well-being in male CAH patients. Sexual function is best measured by patient self-report avoiding interviewer bias and only patients can report on issues such as sexual interest and the extent to which sexual dysfunction has an adverse effect on their quality of life [4]. The Brief Sexual Function Inventory (BSFI) provides an excellent tool to assess a self-reported measure of current sexual functioning [4].

The aims of our two-year prospective study in adult male patients with congenital adrenal hyperplasia were

1. to investigate changes in hypothalamic-pituitary-testicular regulation by GnRH testing,
2. to evaluate changes in sexual functioning and quality of life.

## 2. Methods

### 2.1. Patient Population

The subjects were adult male patients with confirmed classical CAH due to 21-hydroxylase deficiency with regular hormonal follow-up at the outpatient clinic of the Department of Endocrinology of the Charité Campus Mitte Hospital, Berlin. The study was approved by the ethics committee of the Charité Campus Mitte Berlin (permit no. ES1/037/06). All subjects gave written informed consent. For individual patient characteristics, see Table 1. Exclusion criteria were other diseases with impairment of gonadal capacity and other general and psychiatric diseases.

All patients were seen at the outpatient clinic by two experienced endocrinologists (M.Q.; M.V.) on a regular basis every six months. In each patient physical examination, blood drawings (between 0800 and 1000 h, 2 h after morning medication), questionnaires, and testicular ultrasounds were performed at study start (baseline) and two years later (follow-up). The treating physicians tried to optimize treatment during the study period according to the Endocrine Practice guidelines [5].

The standard medications for the treatment of 21-OHD deficiency are hydrocortisone (HC), prednisolone (PR), and dexamethasone (DX) [6, 7]. Since these glucocorticoids have different biological strengths, dosage for PR and DX were converted into hydrocortisone equivalent (PR was converted 1 to 5 to HC, DX 1 to 70 to HC) [8, 9]. After conversion to the hydrocortisone equivalent dose, the daily total amount of hydrocortisone equivalent in milligrams was calculated as well as the total daily dose per body surface area (mg/m^2^).

### 2.2. Testicular Imaging

Grey-scale and color Doppler ultrasonography of the testes was obtained in longitudinal and transverse sections.

### 2.3. Hormone Measurements

Circulating concentrations of Δ4-androstenedione (AD) (BeckmannCoulter, Krefeld, Germany), testosterone, DHEAS (DPC Biermann GmbH; Bad Nauheim, Germany), LH, FSH, renin concentrations, ACTH, and 17-hydroxy-progesterone (17-OHP) (MP Biomedicals GmbH; Eschwege, Germany) were measured by commercially available assays. The GnRH stimulation test was performed by administering 100 *μ*g GnRH (Aventis Pharma GmbH, Frankfurt, Germany) as an i.v.bolus. Serum FSH and LH levels were measured at 0 and 30 min after GnRH dose. We used the differences between peak and basal LH and FSH concentrations, referred to as Δmax⁡, as response variables to eliminate the additive effect of basal LH or FSH level on the peak [10]. A normal LH response in GnRH testing was assumed if LH levels rise was at least 3-fold; normal FSH response if FSH rises was at least 50% or above 3 IU/L.

### 2.4. Questionnaires

Patients were asked to complete four different questionnaires. Psychometric evaluation of patients was performed using three validated self-assessment subjective health status (SHS) questionnaires: the SF-36, the brief form of the Giessen Complaint List (GBB-24), and the “Hospital Anxiety and Depression Scale” (HADS). In addition, the sexual functioning was assessed by the male Brief Sexual Function Inventory (BSFI). All four questionnaires were presented as self-explanatory, multiple-choice self-assessments.

The SF-36 questionnaire is the most widely used generic instrument to assess Quality of Life (QoL) [11]. It consists of eight multi-item domains representing physical functioning (PF), role functioning physical (RP), bodily pain (BP), general health perception (GH), vitality (VT), social functioning (SF), role functioning emotional (RE), and mental health (MH). The domain scores range from 0 to 100 with higher values indicating better QoL [12, 13].

The HADS measures in 14 items anxiety and depression in physically ill individuals [14]. Each item is scored as a number, with a maximum score of 21 for each subscale. Higher scores indicate higher levels of anxiety or depression. A cut-off value of 8 is regarded as indicating mild impairment, and a cut-off value of 11 is indicative of severe impairment.

The short form of the GBB-24 questionnaire consists of 24 items defining four subscales (exhaustion tendency, gastric symptoms, pain in the limbs, and heart complaints), each including six items with ratings from 0 to 4. In addition, a global score of discomfort (GSD) is calculated by adding the four subscale scores. The maximum value for each subscale is 24, and for the global score 96. Higher scores indicate greater impairment of well-being [15].

Regarding control group data we calculated the *z*-scores by using reference data for SF-36 scores obtained from the German National Health Survey (Bundesgesundheits-Survey 1998, Robert Koch Institut, Berlin 2000, public use file BGS 98) comprising a representative random sample of 7124 subjects from the German population aged between 18 and 79 yr. Reference data for the HADS (*n* = 4410) and the GBB-24 (*n* = 2076) were obtained from previously performed surveys [15–17].

The Brief Sexual Function Inventory (BSFI) was used to assess perceived problems associated with sexual drive (two items), erection (three items), problem assessment (three items), ejaculation (two items), or overall satisfaction (one item). Each question was scored on a 5-point scale, ranging from 0 to 4, with lower scores indicating worse sexual function [4]. This questionnaire is useful for measuring male sexual function in practice and research. Regarding control group data we calculated the *z*-scores by using normative data for the BSFI obtained from a representative random sample of 1185 subjects from the Norwegian population aged between 20 and 79 yr [18].

### 2.5. Statistical Analysis

Variables were assessed for normality by Kolmogorov-Smirnov test. Results are expressed as mean ± standard deviation (SD) if not stated otherwise. The significance of data was determined by Students *t* test in normally distributed and in not normally distributed data by Mann-Whitney-Wilcoxon test where appropriate. In addition, Pearson's correlation was performed. A *P* value less than 0.05 was regarded as significant. Statistical analysis was performed using SPSS for Windows 15.0 (SPSS, Inc., Chicago, IL).

## 3. Results

At baseline 28 adult male patients with CAH were screened for study inclusion. Eight patients were excluded due to testicular operations or other exclusion criteria. Finally 20 patients were enrolled into the study. Three patients did not participate in the 2-year follow-up visit and were not included into the 2-year follow-up analysis.

Clinical and genetic characteristics are shown in Table 1: 14 patients had salt-wasting CAH and 6 patients had simple-virilizing CAH. The mean age was 28.9 ± 10.5 years. Patients with salt-wasting CAH were diagnosed within the first week after birth; patients with simple-virilizing form were diagnosed in the first 7 years after birth.

Biochemical and hormonal parameters of the 17 patients at baseline and at the 2-year follow-up visit are presented in Table 2. Over the study period BMI, systolic blood pressure, lipids, androgens, and androgen precursors did not change significantly in the whole cohort or in the SW and SV subgroups. Only diastolic blood pressure was significantly lower at the 2-year follow-up. The daily HC equivalent dose did not decrease significantly. Information about fatherhood was available in 16 of the 17 men studied. Of these, two were fathers (12.5%), one of one child and the other of two children.

### 3.1. Adrenal Crisis

During the study period three adrenal crises occurred resulting in a calculated incidence of 8.8 adrenal crises per 100 patients/year, which is higher than the recently reported frequency in CAH patients (4.8 crises per 100 patients/year) [19] and resembles more the frequency in patients with primary adrenal insufficiency (6.6 crises per 100 patients/year) [20].

### 3.2. Hormonal Control

Decreased DHEAS levels were measured in 15 patients (88.2%) at baseline and in all patients at follow-up (100%). Poor therapy control with elevated serum 4-androstenedione levels (>10 nmol/L) was observed in no patients at baseline (0%) and in only one patient (5.9%) at follow-up, whereas elevated levels of 17-OHP in serum (>36 nmol/L) were present in 4 patients (23.5%) at baseline and 2 patients at follow-up (11.8%). The androstenedione to testosterone (AD/T) ratio as indicator of testicular testosterone production was normal (<0.2) in 11 patients (64.7%) at baseline and follow-up; three patients (17.6%) had an AD/T ratio > 1 suggesting testosterone from predominantly adrenal origin (Table 2). Estradiol levels were within the normal male range ruling out any suppression of the hypothalamus-pituitary-gonadal axis by estradiol.

### 3.3. Gonadotropic Axis

Total testosterone levels were decreased in 4 patients (23.5%) at baseline and in 2 patients (11.8%) at follow-up; calculated free testosterone index was diminished in 5 patients (29.4%) at baseline and in 8 patients (47.1%) at follow-up. Basal LH levels were normal (1.5–9.3 IU/L) in all patients at baseline and in all but one patient at follow-up. Basal FSH levels were elevated in three patients at baseline (17.6%) and normal in all patients at follow-up.

GnRH stimulation induced an adequate increase in LH in all but one patient at baseline (5.9%) and in all but two patients (11.8%) at follow-up. FSH failed to increase sufficiently by GnRH stimulation in two patients at baseline and at follow-up (11.8%).

Three patients (18%) showed TART in testicular ultrasound with a size of 6–11 mm. In one patient TART regressed and was not detected after 2 years. In a subset of patients (6 of the 17 patients) we were able to perform a semen analysis, which showed a pathological status.

Patients with an AD/T ratio below 0.2, indicating sufficient adrenal suppression and a testosterone of testicular origin, showed significant lower 17-OHP and AD levels than patients with an AD/T ratio > 0.2 (Table 3). Significantly more patients with an AD/T ratio < 0.2 received dexamethasone. Basal LH and FSH levels as well as testosterone levels were not different between the groups. However, the Δmax⁡ increase in LH in GnRH testing was significantly higher in the patients with an AD/T ratio < 0.2 than those with an AD/T ratio > 0.2 (Table 3). No differences in TARTs or adrenal crisis were found between the groups.

### 3.4. Questionnaires

#### 3.4.1. General Well-Being

**Figure 1:**
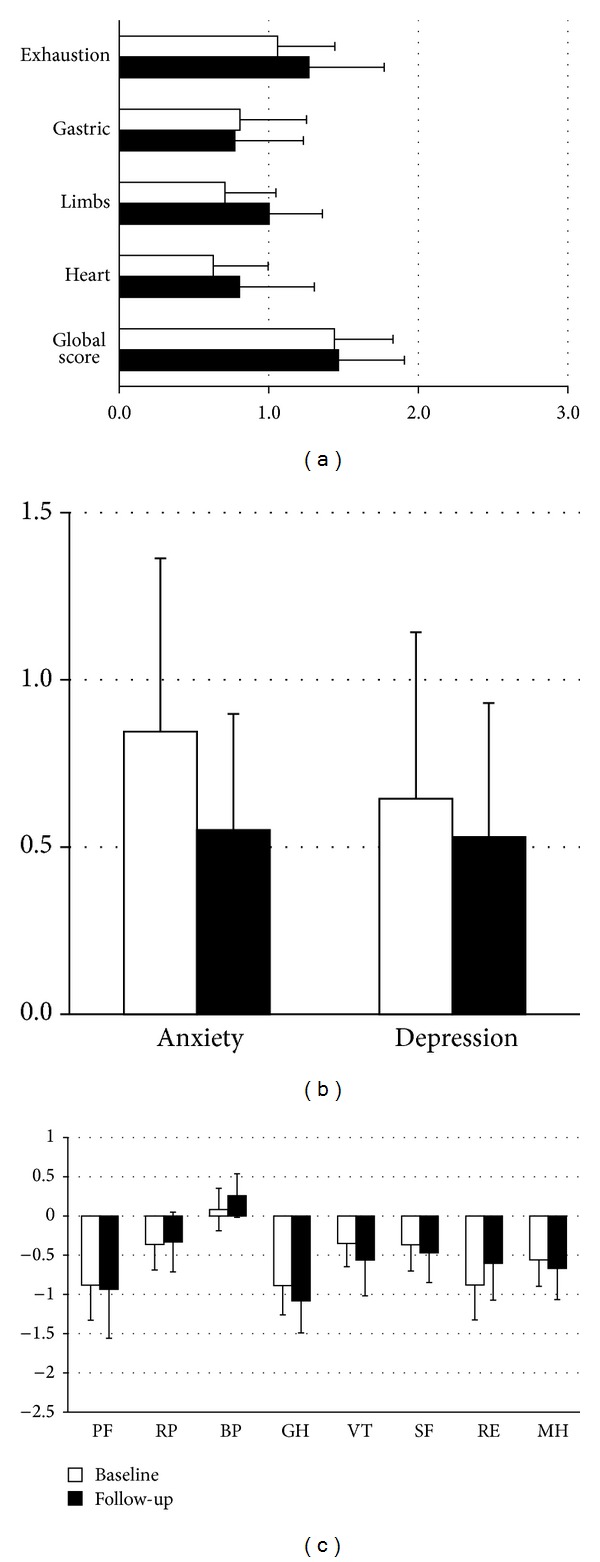
*z*−scores (mean ± SEM scores) for (a) GBB-24, (b) HADS, and (c) SF-36 in male patients with congenital adrenal hyperplasia at baseline and after 2-year follow-up. Decade- and sex-adjusted *z*-scores were calculated for subgroup analysis. Higher scores indicate greater impairment of well-being, anxiety, or depression in GBB-24 and HADS. Higher scores in SF-36 indicate less pain or less impaired functioning. The respective control group has the *z*-score value 0. Physical functioning (PF); role functioning physical (RP); bodily pain (BP); general health perception (GH); vitality (VT); social functioning (SF); role functioning emotional (RE); mental health (MH).

Analysis of the QoL questionnaires (GBB-24, HADS, and SF-36) revealed no significant changes in *z*-scores during the 2-year study period in our adult male CAH patient cohort (Figure 1). However, all dimensions of the GBB-24 showed a trend to increased *z*-scores indicating an impairment of QoL (Figure 1(a)). Similar results were found for the anxiety and depression *z*-scores of the HADS questionnaires (Figure 1(b)). *z*-scores of the SF-36 questionnaire showed a trend to impairments especially in the dimensions “physical functioning,” “general health perception,” and “emotional role functioning” (Figure 1(c)). The dimensions role “physical functioning” and “bodily pain” were the least impaired parameters.

#### 3.4.2. Sexual Well-Being

**Figure 2:**
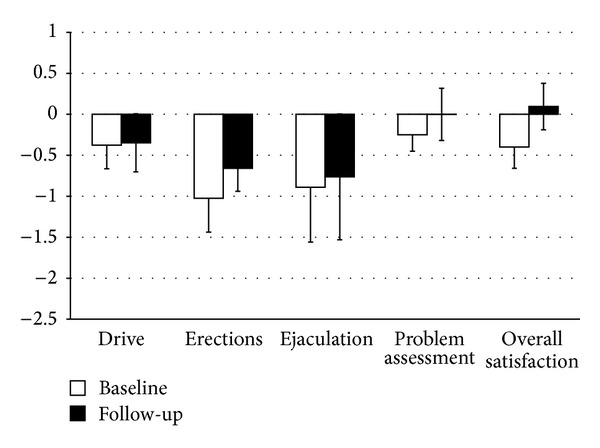
*z*-scores (mean ± SEM scores) for the male Brief Sexual Function Inventory (BSFI) in male patients with congenital adrenal hyperplasia at baseline and after 2-year follow-up. Lower scores indicate higher levels of impairment. The control group has the *z*-score value 0.

The analysis of the participants' *z*-scores revealed that male CAH patients exhibited a slightly reduced sexual drive. More pronounced impairments were observed for the dimensions “erections” and “ejaculations,” whereas the dimensions “problem assessment” and “overall satisfaction” revealed normal *z*-scores (Figure 2). No significant differences in BSFI *z*-scores were found between patients with AD/T ratio < 0.2 or >0.2.

**Figure 3:**
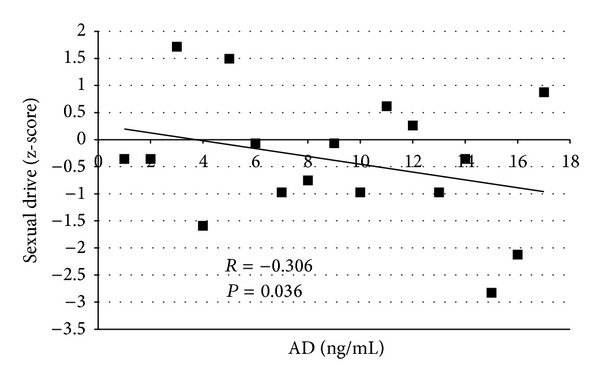
Correlation of androstenedione (AD) levels and *z*-scores of “sexual drive” of the male Brief Sexual Function Inventory (BSFI) in male patients with congenital adrenal hyperplasia at baseline. Higher scores indicate less impaired functioning.

Further analysis showed that AD levels significantly negatively correlated with *z*-scores of the dimension “sexual drive” (*P* < 0.05; Figure 3) with higher AD levels associated with lower *z*-scores (=impaired “sexual drive”).

Decreases in QoL and sexual well-being were not correlated with the presence of TARTs.

## 4. Discussion

Development of TARTs and suppression of the gonadal axis are possible factors that might cause reduced fertility in male CAH patients [1, 2, 21]. It is suggested that adrenal-derived androgen excess due to insufficient hormonal control might be the underlying cause [2, 3]. A recent study in adult male CAH patients revealed a high prevalence of impaired Leydig cell function and impaired spermatogenesis [1]. However, the authors found no correlation between semen parameters, hormonal control, and TART prevalence or size [1].

In our current study, the majority of patients showed basal testosterone and LH within the normal range of young healthy men [10] suggesting normal Leydig cell function in most of the patients. After two years no significant differences were observed in our patients indicating stable therapeutic regimens. However, LH and FSH showed a more pronounced increase (Δmax⁡) after GnRH stimulation than reported in healthy normal males [10]. We further subdivided our cohort into a group with good hormonal control and only testicular testosterone production indicated by an androstenedione/testosterone (AD/T) ratio < 0.2 and a group with poorer disease control and mixed adrenal and testicular testosterone production indicated by an AD/T ratio > 0.2 [22]. The group with an AD/T ratio > 0.2 showed a normal LH and FSH response (Δmax⁡) to GnRH compared to healthy young men [10]. However, the group with an AD/T ratio < 0.2 presented a significant higher LH response to GnRH testing. This resembled a prepubertal response in GnRH testing but might be also due to a suppressed hypothalamic-pituitary axis with a decreased release of GnRH from the hypothalamus. This might be caused by abundant adrenal androgens, which seems not to be the case in this group with AD/T ratio < 0.2. Another explanation might be hypothalamic suppression by glucocorticoids. Interestingly, the percentage of dexamethasone treated patients was significantly higher in the group with an AD/T ratio < 0.2 compared to the group with a ratio > 0.2, but we did not find a significant difference in total daily glucocorticoid equivalent dose per body surface between the two groups. In addition, the amount of glucocorticoid used was approximately similar to that used in other recent studies with male CAH patients [1, 23]. We assume that total glucocorticoid doses were not too high because our patients showed still normal and not suppressed LH levels. In summary, this suggests that dexamethasone has a profound effect on the hypothalamic-pituitary feedback regulation. This is in accordance with previous reports that changing glucocorticoid medication from hydrocortisone to dexamethasone resulted in an increased fertility [24].

Besides these somatic causes of impaired fertility in CAH males, there might be aspects of psychosocial adaption and sexual well-being which might be additional factors for impaired fertility. We investigated sexual well-being by using the male BSFI. We performed this in a prospective fashion and used also quality of life questionnaires to detect possible other changes or general influences during the 2-year study period. During the study period our CAH patients showed unchanged BMI, unchanged metabolic and hormonal parameters, and unchanged impaired *z*-scores in QoL questionnaires. Impaired QoL in male CAH patients has been shown in previous studies [7, 25–27]; however, these had only cross-sectional and not a longitudinal design. We did not find differences in QoL *z*-scores in patients that were on dexamethasone and prednisolone treatment compared to hydrocortisone only as previously reported [27]. This might be due to the rather small number of patients in our subanalysis.

As for sexual well-being, we detected that especially the dimensions “sexual drive,” “erections,” and “ejaculations” were impaired in our cohort. Interestingly, the dimensions “problem assessment” and “overall satisfaction” revealed normal *z*-scores. It is important to point out that “overall satisfaction” should not be confused with the mean score of the functional domains of the BSFI [18], and additional factors might be involved not covered by the questions. It is known that patients with low scores on functional domains, for example, ejaculatory impairment as a side-effect of an anti-depressant drug, do not necessarily report reduced overall sexual satisfaction [18].

We are the first to describe a clearly impaired sexual well-being in male CAH patients by using an established sexual function questionnaire. Interestingly, we observed that poor disease control, according to elevated androstenedione levels, was associated with a reduced “sexual drive.” This directly links CAH therapy with the aspect of sexual well-being. Therefore, we believe that aspects of psychosocial adaption and sexual well-being might be important additional factors for impaired fertility in our male CAH patients.

There might be additional factors for lower fecundity in male CAH patients. First, CAH patients have had a chronic disease since their childhood, as well as having been exposed to exogenous glucocorticoids also during pubertal development. Secondly, male CAH patients have still a lower height than the average male population [28] and this might cause problems in psychosocial adaptation. However, a recent Hungarian study showed that sexual activity was not clearly related to other anthropometric parameters such as height [29].

Possible limitations of our study are as follows. (1) There is no normative data for Germany for calculating *z*-scores for the BSFI, and we had to rely on normative data from Norway. However, no significant differences in functional BSFI scores were found between the Norwegian data and American data from the Olmsted County [18]. (2) There is increasingly reduced sexual function concerning drive, erection, ejaculation, and problem assessment with age with most of these age-related effects starting at >50 years old [18]. However, our patients were all below the age of 50 y. (3) Our study is a rather small cohort of male CAH patients; however, this is the first longitudinal study in adult male CAH patients.

In conclusion, we showed that male CAH patients with a normal AD/T ratio showed an increased LH and FSH response in GnRH testing indicating possible decreased hypothalamic GnRH release by glucocorticoid therapy. Secondly, we found that male CAH patients had impaired sexual well-being, especially regarding erections, ejaculations, and sexual drive. This might be an additional factor for reduced fecundity in adult CAH male patients.

## Figures and Tables

**Table 1:** Clinical and genetic characteristics of 20 male patients with 21-OHD at study start (baseline).

Pat. no.	Pheno-type	Genotype	Mutation group	Age (yr)	BMI (kg/m^2^)	Height (cm)	GC dose equivalent/m^2^ BSA (mg/m^2^)	FC dose/m^2^ BSA (*μ*g/m^2^)
B02	SW	I2G/I172N	B	23	19.0	178	14.52	58.07
B06	SW	I2G + P453/I2G + P453S	B	24	23.0	164	22.29	63.69
B09	SW	V281L/del + V281L	nc	18	16.5	171	33.33	66.67
B10	SW	I172N/I172N	B	18	26.0	170	15.79	52.63
B12	SW	I2G/I2G	A	32	28.0	184	19.91	88.50
B14	SW	I2G/del	A	25	27.5	177	14.63	48.78
B15	SW	I2G/I2G	A	21	26.5	163	19.44	55.56
B16	SW	I2G/del	A	23	25.0	170	27.78	55.56
B17	SW	I2G/del	A	20	30.0	171	26.83	48.78
B18	SW	I172/del	B	49	25.0	155	20.22	37.37
B20	SW	nd		18	20.0	174	13.24	58.82
B07	SW	del/R356W	Null	23	28.0	172	17.68	50.50
B08	SW	I2G-V281L/del-V281L	A	29	25.5	160	13.24	58.86
B22	SW	I2G/E6 cluster	A	23	25.0	180	20.0	50.00
B01	SV	I2G/I172G	B	42	36.0	158	10.01	
B03	SV	I172N/I172N + del	B	47	27.0	153	16.68	
B04	SV	I2G/172N	B	42	24.0	153	19.61	
B13	SV	I172N/Q318X + V281L	B	26	38.0	172	20.30	
B19	SV	I172N/del	B	47	30.0	162	19.95	
B21	SV	I2G/I172N	B	28	24.0	164	22.59	
SW Mean ± SD				24.7 ± 8.1	24.6 ± 3.8	170.6 ± 8.0	19.92 ± 5.97	56.70 ± 11.68
SV Mean ± SD				38.7 ± 9.3	29.8 ± 6.0	160.3 ± 3.0	18.19 ± 4.43	
All CAH Mean ± SD				28.9 ± 10.5	26.2 ± 5.0	167.6 ± 9.0	19.40 ± 5.50	

SW: classical salt wasting; SV: classical simple virilizing; BMI: body mass index; BSA: body surface area; FC: fludrocortisone. The dose of daily glucocorticoid (GC) was converted into milligrams of daily hydrocortisone equivalent (1 mg dexamethasone = 14 mg prednisolone = 70 mg hydrocortisone). Mutation grouping was done according to Krone et al. [30]; nc: not classified; nd: not done.

**Table 2:** Biochemical and hormonal parameters in 17 male patients with 21-OHD at study start (baseline) and after 2-year follow-up.

	Baseline	Follow-up
BMI (kg/m^2^)	26.2 ± 5.5 (16.5–38)	25.5 ± 5.6 (19–38.5)
BP systolic/diastolic (mmHg)	118/78 ± 12/8 (100–135/60–90)	117/73* ± 17/10 (90–145/60–90)
Potassium (mmol/L)	3.76 ± 0.43 (3.2–4.7)	3.92 ± 0.34 (3.4–4.6)
Sodium (mmol/L)	140.8 ± 1.8 (136–144)	139.5 ± 2.7 (134–143)
Cholesterol (mg/dL)	169 ± 36 (126–237)	185 ± 43 (106–247)
Triglycerides (mg/dL)	103 ± 57 (44–219)	116 ± 62 (48–256)
ACTH (pg/mL)	153 ± 331 (5–1250)	62 ± 141 (5–561)
17-OHP (ng/mL)	14.9 ± 18.8 (0.8–50)	25.2 ± 58.8 (0.8–218)
Androstenedione (ng/mL)	1.55 ± 2.39 (0.2–10)	1.85 ± 3.15 (0.2–10.4)
DHEAS (ng/mL)	620 ± 778 (90–3395)	442 ± 378 (1–1246)
Testosterone (ng/mL)	5.0 ± 2.6 (1.7–9.6)	4.4 ± 2.6 (1.5–9.5)
SHBG (nmol/L)	41 ± 20 (14–77)	44 ± 25 (12–99)
Free testosterone index	46.3 ± 20.5 (15.4–100.6)	40.1 ± 24.4 (7.3–113.8)
AD/T ratio	0.41 ± 0.57 (0.06–1.96)	0.45 ± 0.81 (0.06–3.26)
Estradiol (pg/mL)	26.6 ± 8.0 (15.6–41.1)	21.8 ± 12.2 (5.0–49.5)
Renin (ng/L)^§^	84.3 ± 130.7 (2.5–330)	288.7 ± 767.7 (2.5–2592)
LH basal (U/L)	4.1 ± 2.2 (1.5–8.9)	3.8 ± 2.9 (0.6–7.9)
LH peak (U/L)	31.9 ± 12.3 (13.5–61.3)	25.7 ± 16.8 (0.8–53.4)
Δmax LH	27.8 ± 11.1 (7.9–53.6)	22.0 ± 15.5 (0.3–46.2)
FSH basal (U/L)	7.1 ± 6.8 (2.3–28.9)	6.1 ± 4.0 (1.9–12.9)
FSH peak (U/L)	12.9 ± 11.1 (4.4–46)	9.5 ± 8.5 (1.0–26.2)
Δmax FSH	5.9 ± 4.8 (0.6–17.1)	4.3 ± 4.5 (1.0–13.3)
Daily GC equivalent dose/m^2^ BSA (mg/m^2^)	19.8 ± 5.8 (10.0–33.3)	18.6 ± 6.9 (9.2–30.6)

Data are means ± SD (range). BMI: body mass index; BP: blood pressure; BSA: body surface area. Normal ranges (SI units shown in brackets): sodium 134–145 mmol/L; potassium 3.4–5.2 mmol/L; cholesterol < 200 mg/dL (5.17 mmol/L); triglycerides < 180 mg/dL (2.06 mmol/L). Conversion factors: androstenedione (AD) X3.49 nmol/liter; testosterone (T) X3.47 nmol/liter; 17-hydroxy-progesterone (17OHP) X3.026 nmol/liter; estradiol X3.67 pmol/liter; and DHEAS X2.57 nmol/liter. Δmax denotes the differences between peak and basal LH or FSH concentration. The dose of daily glucocorticoid was converted into milligrams of daily hydrocortisone equivalent (1 mg dexamethasone = 14 mg prednisolone = 70 mg hydrocortisone). Free testosterone index (fTI) was calculated by the ratio 347 ∗ testosterone (ng/mL)/SHBG (nmol/L) [31]. ^§^Renin was measured only in SW CAH patients. *P < 0.05 versus baseline.

**Table 3:** Biochemical and hormonal parameters in male patients with 21-OHD depending on AD/T ratio at study start (baseline).

	AD/T ratio <0.2	AD/T ratio >0.2
BMI (kg/m^2^)	25.4 ± 5.9	27.8 ± 4.8
BP systolic/diastolic (mmHg)	120/79 ± 14/7	119/79 ± 6/6
Potassium (mmol/L)	3.81 ± 0.36	3.68 ± 0.55
Sodium (mmol/L)	141.2 ± 0.9	140.0 ± 2.8
Cholesterol (mg/dL)	178 ± 40	154 ± 22
Triglycerides (mg/dL)	106 ± 68	100 ± 44
17-OHP (ng/mL)	2.7 ± 2.5	35.2 ± 16.1***
Androstenedione (ng/mL)	0.52 ± 0.27	3.28 ± 3.36*
DHEAS (ng/mL)	425 ± 239	944 ± 1228
Testosterone (ng/mL)	5.7 ± 2.6	4.0 ± 2.4
Free testosterone index	47.0 ± 12.5	33.1 ± 17.4
AD/T ratio	0.09 ± 0.03	0.94 ± 0.68***
Estradiol (pg/mL)	25.5 ± 8.3	28.2 ± 3.4
LH basal (U/L)	4.4 ± 2.4	3.6 ± 1.9
Δmax LH	31.5 ± 10.1	18.5 ± 8.1*
FSH basal (U/L)	8.1 ± 7.9	4.5 ± 1.3
Δmax FSH	7.1 ± 5.1	2.8 ± 1.9
No. of adrenal crisis during 2-year follow-up	2	1
% of patients with TART	18%	17%
daily GC equivalent dose/m^2^ BSA (mg/m^2^)	20.5 ± 5.7	18.7 ± 6.2
% of patients receiving any treatment regimen with dexamethasone	55%	0%*
% SW	64%	67%
Daily fludrocortisone dose/m^2^ BSA (mg/m^2^), ^§^	60.1 ± 15.4	53.5 ± 7.1

Data are means ± SD. BMI: body mass index; BP: blood pressure; BSA: body surface area. Normal ranges (SI units shown in brackets): sodium 134–145 mmol/L; potassium 3.4–5.2 mmol/L; cholesterol < 200 mg/dL (5.17 mmol/L); triglycerides < 180 mg/dL (2.06 mmol/L). Conversion factors: androstenedione (AD) X3.49 nmol/liter; testosterone (T) X3.47 nmol/liter; 17-hydroxy-progesterone (17OHP) X3.026 nmol/liter; estradiol X3.67 pmol/liter; and DHEAS X2.57 nmol/liter. Δmax denotes the differences between peak and basal LH or FSH concentration. The dose of daily glucocorticoid was converted into milligrams of daily hydrocortisone equivalent (1 mg dexamethasone = 14 mg prednisolone = 70 mg hydrocortisone). Free testosterone index (fTI) was calculated by the ratio 347 ∗ testosterone (ng/mL)/SHBG (nmol/L) [31]. ^§^Only in SW CAH patients. *P < 0.05; ***P < 0.001.
